# A novel stress response mechanism, triggered by indole, involved in quorum quenching enzyme MomL and iron-sulfur cluster in *Muricauda olearia* Th120

**DOI:** 10.1038/s41598-017-04606-8

**Published:** 2017-06-26

**Authors:** Yan Wang, Hui Li, Xinxin Cui, Xiao-Hua Zhang

**Affiliations:** 1 College of Marine Life Sciences, Ocean University of China, Qingdao, 266003 China; 2 Laboratory for Marine Ecology and Environmental Science, Qingdao National Laboratory for Marine Science and Technology, Qingdao, 266071 China

## Abstract

Indole, as a signal molecule, is involved in multiple physiological behavior including biofilm formation, antibiotic resistance and virulence. In this study, we demonstrated that indole was involved in iron deficient and H_2_O_2_ stress response in *Muricauda olearia* Th120. Transcriptome analysis showed that totally 206 genes were regulated by exogenous indole. Besides, *momL-suf* gene cluster, consisting of quorum quenching enzyme coding gene *momL* and iron-sulfur biosynthetic genes *suf*, were involved in indole-induced stress response pathway. The result indicated that indole not only up-regulated *momL-suf* gene cluster, but also enhanced the MomL secretion and the growth rates of MomL-bearing strains in H_2_O_2_ stress and iron deficient culture conditions. Co-incubation of *M. olearia* Th120 and *Pectobacterium carotovorum* subsp. *carotovorum* under H_2_O_2_ condition revealed that *M. olearia* Th120 bearing MomL possessed an increased competitive advantage, whereas its competitor had a reduced survival. The phenomenon that quorum quenching enzyme is triggered by stress factor has been rarely reported. The study also opens a new clue to explore the indole function towards quorum quenching factor in bacteria.

## Introduction

Quorum sensing (QS) is a population-dependent behavior that enables bacteria to communicate with each other and to regulate downstream gene expression in response to environmental changes^1–5^. During growth, bacteria produce a type of small molecule known as an autoinducer (AI), which can bind to receptor proteins and trigger signal pathway cascades when the AI concentrations reach a threshold value. At present, several types of AIs have been discovered, such as the *N*-acyl homoserine lactones (AHLs) common in many Gram-negative bacteria^1, 6^, oligopeptides in Gram-positive bacteria and AI-2 in both Gram-negative and Gram-positive bacteria^7–9^. Indole is another signal molecule which has recently received increased attention as a putative QS signal molecule^10, 11^. Many Gram-positive and Gram-negative bacteria encode a single copy of the *tnaA* gene in their chromosome and produce indole. It plays diverse biological roles including biofilm formation, sporulation and virulence^10, 12–14^. It has also been shown that indole, via two component signal transduction systems, increases antibiotic resistance by inducing exporter and transporter genes in *E. coli* ^10^. Hence, it is possible that these two-component signal systems can be used as indole sensors. However, the indole signaling pathway and its function in stress response have not been comprehensively studied, especially in beneficial microorganisms.

Previous studies have demonstrated that QS systems are closely related to bacterial disease. Interfering with QS systems, named quorum quenching (QQ), is a promising control strategy for bacterial diseases because it could decrease the expression of virulence factors of pathogens^15^. Normally, virulence factor is the weapon for pathogens during adverse situation, so theoretically, QQ could weaken pathogens. The marine bacteria *Muricauda olearia* Th120 exhibits strong QQ activity, which makes this strain a potential biocontrol agent^16^. In our previous study, a novel AHL lactonase, MomL, was identified from *M. olearia* Th120^17^. MomL belongs to the metallo-β-lactamase superfamily and shows the highest identity (56.8%) with Aii20J from *Tenacibaculum* sp. 20J^18^. Besides, MomL possesses 54.4% and 24.5% identity with FiaL and well-studied AiiA from *Flaviramulus ichthyoenteri* Th78T and *Bacillus* sp. 240B1 respectively^19–21^. As a novel AHL lactonase in marine isolates, MomL shows promising activity against C6-homoserine lactone (C6-HSL), reaching a catalytic efficiency of 2.9 × 10^5^ s^−1^M^−1^.

Iron-sulfur (Fe/S) proteins, characterized by the presence of an iron-sulfur cluster, play crucial roles in multiple cellular processes in both prokaryotic and eukaryotic cells^22^. The biogenesis of iron-sulfur clusters has been extensively studied in *Escherichia coli* ^22, 23^, in which there are two iron-sulfur cluster biosynthesis gene operons: the ISC (iron-sulfur cluster) and Suf (sulfur assimilation) systems^23^. ISC genes are organized as *iscSUA-hscBA-fdx*, whereas Suf genes are organized as *sufABCDSE*. The ISC gene cluster is thought to play housekeeping roles, including the formation of a variety of Fe/S proteins, whereas the Suf system is active under stress conditions, such as iron starvation^22, 23^.

Interestingly, *momL* is located within the stress-response *suf* operon and is closely linked to *sufB* and *sufC*. This gene organization has not been previously reported in other organisms. Also, little is known about how the QQ factor *momL* is regulated by QS in *M. olearia* Th120. In this study, we focused on the regulation mechanism of signal molecular indole towards the stress-response genes *suf* and QQ gene *momL*, and it firstly described the triggering of the expression of a QQ enzyme by stress factors.

## Material and Methods

### Bacterial strains, media and growth conditions

*M. olearia* Th120 was cultured in marine broth 2216 at 28 °C. The AHL biosensor *Chromobacterium violaceum* CV026 was cultured in LB medium at 28 °C^17^. Indole (Sigma-Aldrich, St. Louis, Missouri, USA) stock solutions were prepared in methanol, and the 2,2′-dipyridyl (Solarbio) stock solution was prepared in ethanol.

### H_2_O_2_ sensitivity assay

*M. olearia* Th120 was grown in marine broth 2216 at 28 °C overnight. The cultures were then diluted with fresh medium to a final optical density at 600 nm (OD600) of 0.1. The cell suspensions were mixed with H_2_O_2_ or H_2_O_2_ with 0.2 mM indole at final concentrations of 0.5, 1 or 2 mM and were incubated at 28 °C for 20 min. The cells were serially diluted and spread onto marine agar plates, and the colony numbers were counted from each of the plates after 48–72 hours of incubation^24^.

### RNA extraction, reverse transcription PCR, and real-time-PCR

*M. olearia* Th120 cells were collected after reaching an OD600 of 0.8, and the RNA was extracted using an RNA extraction kit (Omega) according to the manufacturer’s instructions. For indole treatments, samples with different cultivation conditions were harvested as described above. For the H_2_O_2_ treatment assay, mid-log-phase cultures of *M. olearia* Th120 were harvested. After a 30-min treatment with 300 μM H_2_O_2_, the RNA was extracted and was reverse-transcribed to cDNA. Quantitative real-time PCR was performed in a total reaction volume of 20 μl containing 250 nM primers, 10 μl of SYBR green PCR master mix, 8.5 μl of RNase-free water, and 0.5 μl of a 10-fold-diluted cDNA template. The 16S rRNA was used as a reference gene^25^. Relative gene expression level was analyzed using 2^−ΔΔCt^ method (ΔΔCt = ΔCt_(test)_ − ΔCt_(calibrator)_, ΔCt_(test)_ = Ct_(target, test)_ − Ct_(reference, test)_ and ΔCt_(calibrator)_ = Ct_(target, calibrator)_ − Ct_(reference, calibrator)_). The primers for real-time PCR are listed in Table S1. Real-time PCR was performed with a StepOne Real-Time PCR System (AB Applied Biosystems). The program was designed as follows: pre-incubation at 95 °C for 10 min followed by 35 cycles of 15 seconds at 95 °C, 30 seconds at 55 °C and 30 seconds at 72 °C. After 2 hours, cycle threshold (CT) values and melting curves of each reaction were analyzed using StepOne Software v2.2.

### Extracellular secretion detection of MomL

*M. olearia* Th120 cells were grown for 48 hours, and the OD600 values of the samples (treated and untreated) were measured using a spectrophotometer. The cultures were diluted with fresh medium to the same OD600 value. Filter-sterilized (0.22 μm pore size) supernatant and cells were collected, followed by a 10-min centrifugation at 4 °C at 6000 × g. The AHL biosensor *Chromobacterium violaceum* CV026^26^, which could produce violacein induced by AHL, was cultured on LB medium at 28 °C to an OD600 of 0.1, and 1 mL of culture and C6-HSL at a final concentration of 0.5 μM were added to solid LB medium. The extracellular secretions of samples from different culture conditions were added to the medium, which was placed in an incubator at 28 °C. After 24 hours of culture, transparent circles were observed if AHL was degraded and violacein synthesis was inhibited. The area of transparent circle was calculated and the mean value of three repeated experiment was used.

### Bioinformatic analysis

Protein domains were analyzed by SMART software (http://smart.embl-heidelberg.de/smart/set_mode.cgi?NORMAL=1) for online analysis. The *suf* and *momL* sequences of the different strains were analyzed by BLAST (http://blast.ncbi.nlm.nih.gov/Blast.cgi). Annotation and bioinformatic analysis were performed on the flanking genes of *momL* by genome sequencing and EMBOSS (The European Molecular Biology Open Software Suite) (http://emboss.open-bio.org/). DNAMAN software was used to compare the three copies of the OxyR protein. Primers for real-time fluorescent quantitative PCR were designed using Primer Premier 5^27^.

### Co-cultures of *Pectobacterium carotovorum* subsp. *carotovorum (Pcc)* and *E. coli*

The two types of bacteria were cultured in LB medium separately, and cells were collected by centrifugation when the OD600 reached 0.8. The two types of bacteria were mixed and cultured in 10% tryptic soy broth (TSB) medium with 2 mM H_2_O_2_ for 6 hours. The bacterial co-culture was serially diluted. At each concentration, dilutions were spread onto two LB plates, one of which contained 25 µg/mL Km. The survival of *Pcc* was the difference between the bacterial counts of the two LB plates (Numbers_LB_ − Numbers_LB+Km_).

### Co-cultures of *P. carotovorum* subsp. *carotovorum (Pcc)* and *M. olearia* Th120

*Pcc* and *M. olearia* Th120 were cultured in LB and MA media^28, 29^ separately until the OD600 reached 0.8. Cultures of the two bacteria were centrifuged, and the two types of cells were collected and mixed together. Then, the mixture was inoculated into 10% TSB medium containing 2 mM H_2_O_2_ and cultured for 6 hours. During the culturing, various concentrations of MomL protein were added into the medium. After culturing, the cultures were serially diluted and spread onto MA and LB plates, separately (MA is the regular medium for marine bacterium *M. olearia* Th120, and LB is for *Pcc*. Besides, *M. olearia* Th120 can not survive on LB medium, while, *Pcc* can not live on MA medium). Subsequently, changes in the survival of the two bacteria in the different conditions were observed.

### Transcriptome sequencing and analysis

Transcriptome sequencing of *M. olearia* Th120 (treated with and without indole) was performed by the Biozeron company in Shanghai, China. Total RNA of *M. olearia* Th120 was extracted using TRIzol reagent (Invitrogen) and treated with DNase I (Takara). RNA quality was determined using a Bioanalyser 2100 (Agilent), after which the RNA was quantified using a NanoDrop 2000. RNA transcriptome libraries were constructed following a TruSeq RNA sample preparation kit from Illumina (San Diego, CA), using 5 μg of total RNA. rRNA was removed using a RiboZero rRNA removal kit (Epicenter) and fragmented with fragmentation buffer. cDNA synthesis, end repair, A-base addition and ligation of the Illumina-indexed adaptors was performed following Illumina’s protocol. Libraries were then size selected for cDNA target fragments of 200–300 bp on 2% Low Range Ultra Agarose followed by PCR amplified using Phusion DNA polymerase (NEB) for 15 PCR cycles. After generating the clusters, library sequencing was performed on an Illumina Hiseq platform, to create paired-end reads with lengths of 150 bp. The raw paired-end reads were trimmed with SeqPrep (https://github.com/jstjohn/SeqPrep) and quality controlled with Sickle (https://github.com/najoshi/sickle). Then, clean reads were aligned to the reference genome using Rockhopper (http://cs.wellesley.edu/~btjaden/Rockhopper/). Rockhopper was a user-friendly software for bacterial RNA-seq data anaylsis. This software was used to calculate gene expression level as RPKM (reads per kilobase transcriptome per million mapped reads) with default parameters. RPKM = (10^6^ * C)/(N * L), with C = Number of reads mapped to a gene; N = Total mapped reads in the experiment; L = gene length in base-pairs for a gene (Kb) EdgeR^30^ was used for differential gene expression analysis (https://bioconductor.org/packages/release/bioc/html/edgeR.html). To study the functions of the differentially expressed genes, GO functional enrichment and KEGG pathway analysis were carried out by Goatools (https://github.com/tanghaibao/Goatools) and KOBAS (http://kobas.cbi.pku.edu.cn/home.do) respectively. Gene expression changes higher than 2-fold with P-values less than 0.05 were regarded as reliable and marked differences. The sequencing data were deposited in NCBI Sequence Read Archive with accession number PRJNA375720.

## Results

### Indole improved survival state of *M. olearia* Th120 in H_2_O_2_ stress and iron deficient conditions

**Figure 1:**
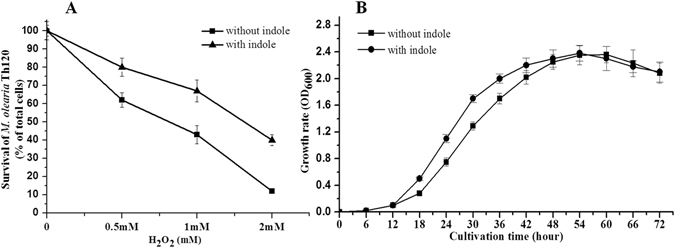
Function of indole for *M. olearia* Th120 in iron deficient and H_2_O_2_ conditions. (**A**) The survival of *M. olearia* Th120 upon H_2_O_2_ treatment with or without indole. (**B**) *M. olearia* Th120 growth rate assay under conditions of ferric infertility (100 μM 2,2′-dipyridyl). Error bars represent the standard deviations of three replicates.

Previous studies indicated that indole is responsible for multiple physiological behaviors, especially in enteric bacteria. Besides, it was reported that indole could leads bacterial persistence formation by activating stress responses^31^. However, the indole function towards stress responses in beneficial bacteria *M. olearia* Th120 is largely unknown. Our experiments indicate that indole can improve the survival of *M. olearia* Th120 in the presence of H_2_O_2_. Specifically, the survival of cells in 0.5, 1, and 2 mM H_2_O_2_ were 80%, 70%, and 45%, respectively, in the presence of 200 μM indole. However, the survival of the cells were 60%, 45%, and 15% in the presence of H_2_O_2_ but the absence of indole (Fig. 1A). Besides, the survival state under iron deficient was also detected. The growth rate assay indicated that 200 μM exogenous indole caused *M. olearia* Th120 to have an improved growth rate in lag and exponential phases under ferric-deficient conditions. No obvious difference was observed after stationary phase. What is more, indole did not affect the cell yield of *M. olearia* Th120 (Fig. 1B and Fig. S1).

### *suf-momL* was involved in indole-induced stress responses in a co-transcriptional manner

In *E. coli*, the *suf* operon is responsible for resistance to iron starvation^23^. We focused on the study of whether *suf* of *M. olearia* Th120 was involved in indole-induced iron deficient and H_2_O_2_ stress response. Interestingly, genome sequencing and bioinformatic studies indicated that the *suf* genes of *M. olearia* Th120 is co-localized within the *momL* gene, an arrangement that has not been reported in similar well-studied genes, such as *aiiA*. The classic *suf* locus contains *sufA, sufB, sufC, sufD, sufS*, and *sufE* (Fig. S2). SufS and SufE are desulfurase homologs, whereas SufC is an ATPase. SufS and SufE are responsible for passing the sulfur that dissociates from cysteine to the Fe-S assembly complex, which is composed of two SufC units and single copies of both SufB and SufD. With the participation of the cofactor FADH_2_, the Suf proteins assemble iron and sulfate into Fe-S clusters. SufA is responsible for transporting Fe-S clusters to the aconitase, AcnB. In previous studies, *suf* was always observed as an intact gene cluster with no other unrelated genes among the *suf* genes. In *M. olearia* Th120*, momL* is inserted between *sufB* and *sufC*, and these three genes are linked closely to each other.

**Figure 2:**
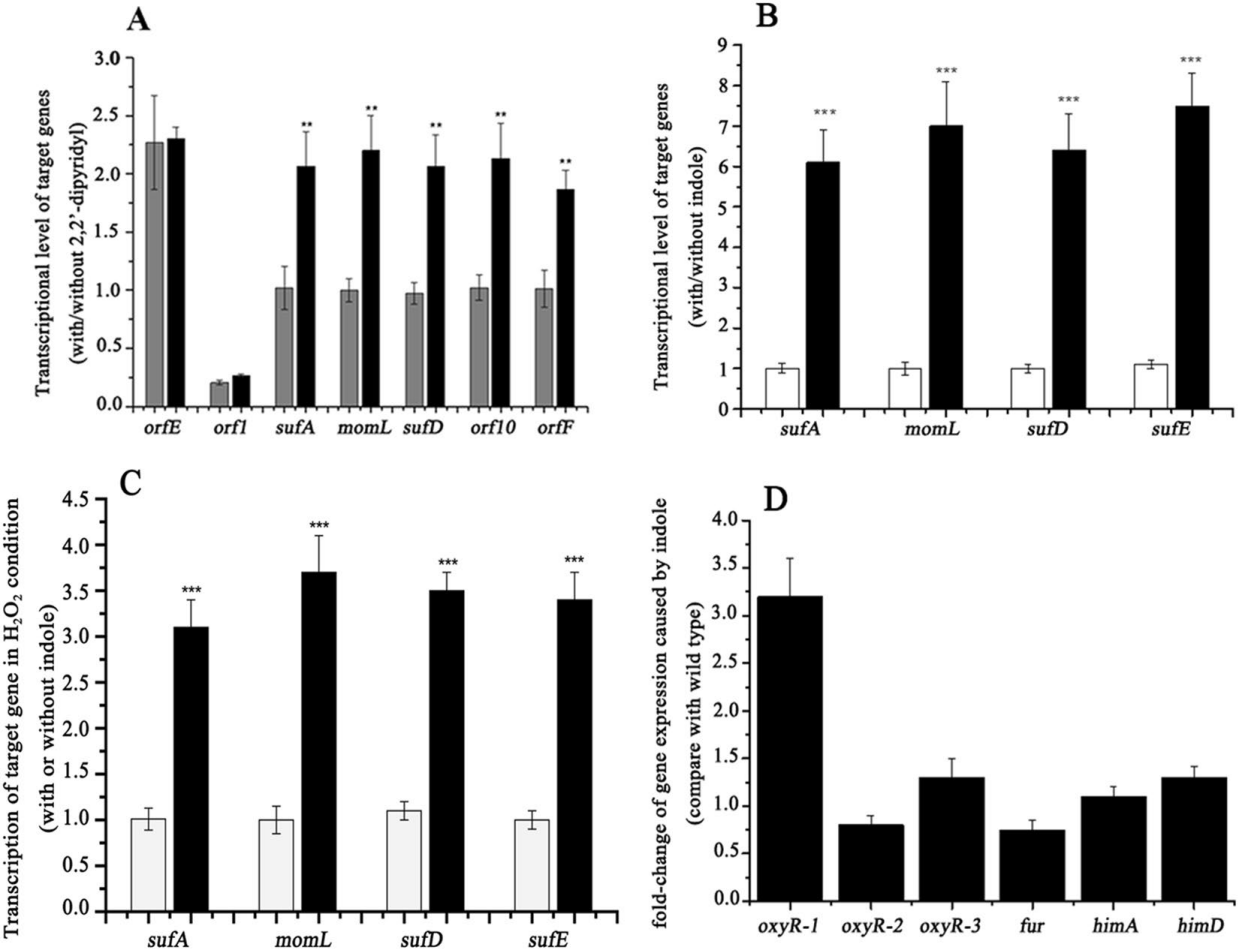
The transcriptional analysis (Ct values) of related genes. (**A**) Transcription levels of genes in the *suf*-*momL* operon with (black) or without (gray) the iron-chelating agent 2,2′-dipyridyl. (**B**) Transcription levels of *suf-momL* genes in iron-deficient condition with (black) or without indole (transparent). (**C**) indole-induced transcriptional change in H_2_O_2_ condition (black, with indole; transparent, without indole). (**D**) The effects of indole on a series of *suf* regulators. Error bars represent the standard deviations of three replicates. For statistical analysis, ***/**/* means P < 0.001, P < 0.01, and P < 0.05, respectively.

To analyze the transcriptional features of *momL* and *suf*, *orfE*/*orf1*/*sufA*/*momL*/*sufD*/*orf10* and *orfF* were chosen for real-time PCR analysis. In normal condition, the *sufA*, *momL*, *sufD*, *orf10* and *orfF* transcription levels were highly similar, whereas the transcript level of *orfE*, whose expression was approximately 2-fold higher compared with that of *sufA*, differed greatly from the expression level of *sufA*-*orfF*. Moreover, the expression level of *orf1* was very low: only 20% of that of *sufA* (Fig. 2A). These differences suggest that *suf* and *momL* are co-transcribed, whereas *orf1* and *orfE* are expressed independently of *suf-momL*. In addition, *orf10*, which encodes a novel Fe-S cluster synthesis protein, and *orfF*, which encodes an unknown protein, are also co-transcribed with *suf-momL*.

*suf-momL* was involved in indole-induced stress responses pathway. The transcription of *suf-momL* was analyzed under indole-induced iron-deficient and H_2_O_2_ condition. Without indole, the *sufA*, *sufD*, *orf10* and *orfF* expression levels were approximately 2-fold higher in an environment containing the intracellular iron (II) chelator 2,2′-dipyridyl (100 μM), whereas the *orfE* and *orf1* expression levels were not significantly altered in this iron-deficient condition (Fig. 2A). While, with 200 μM indole, the transcription levels of *sufA*, *sufD*, and *sufE* increased 6-, 6-, and 7-fold, respectively, and the transcription level of *momL* increased nearly 7-fold in this iron-deficient condition (Fig. 2B). Similarly, in H_2_O_2_ stress condition, indole up-regulated *sufA*, *sufD*, *sufE* and *momL* to 3–4 fold changes respectively (Fig. 2C). Previous studies have indicated that three proteins are involved in regulating the *suf* genes: OxyR, a peroxide-sensing positive regulator; Fur, a ferric uptake regulator; and HimA/D, a bacterial nucleoid DNA-binding protein that induces increased *suf* expression during oxidative stress^23, 32, 33^. A bioinformatic analysis of *M. olearia* Th120 revealed that the genome includes not only the *fur* and *him* genes but also three copies of the *oxyR* gene, designated *oxyR1/2/3*. These *oxyR* genes contain well-conserved DNA-binding sites (Fig. S3). Transcriptional analysis showed that there were no marked changes in the levels of *oxyR2*, *oxyR3*, *fur*, or *himA* in the presence of indole, whereas *oxyR1* expression increased significantly at 3.2-fold over normal conditions (Fig. 2D).

**Figure 3:**
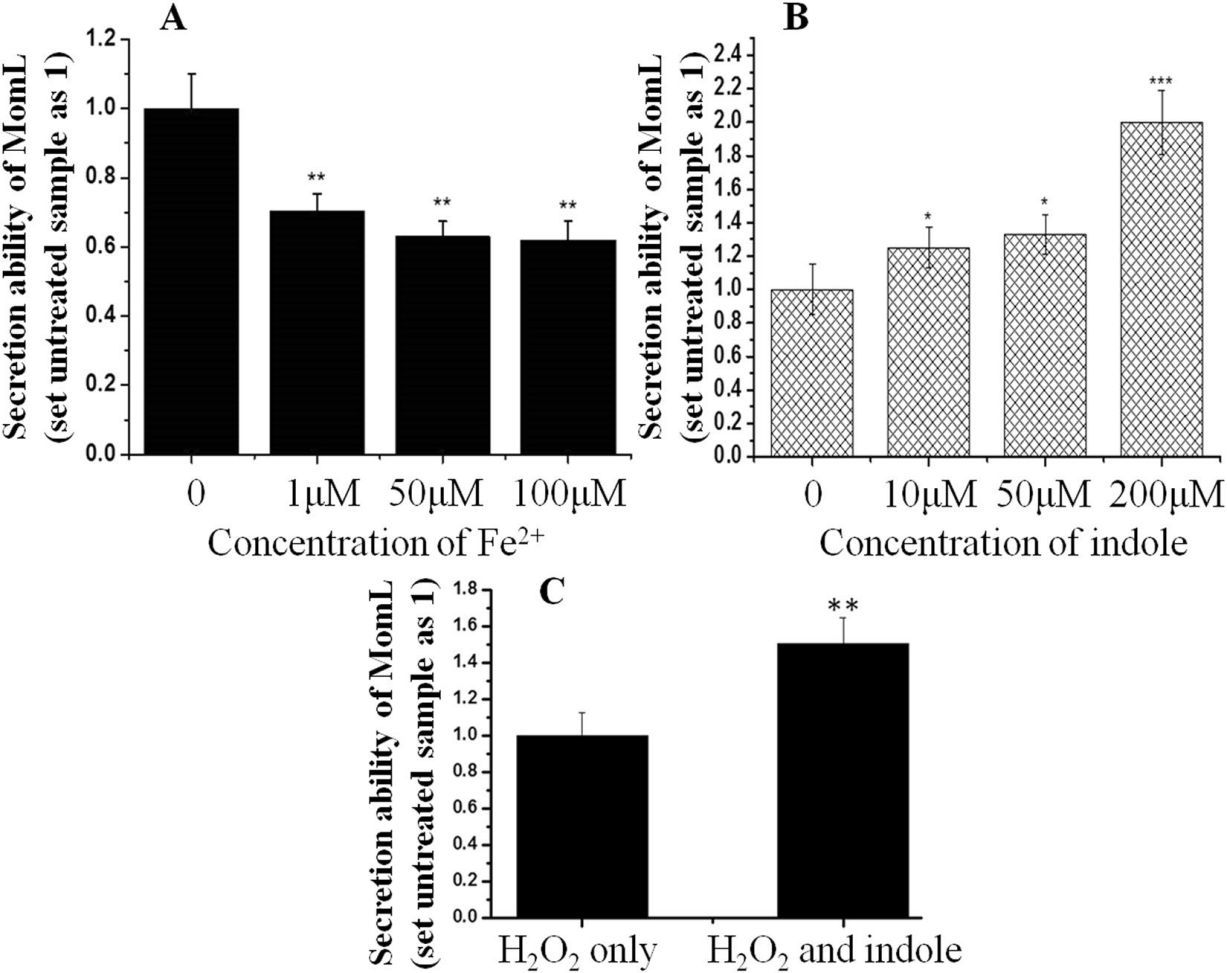
MomL secretion capacity analysis. (**A**) The secretion capacity of MomL in different iron environments. (**B**) Indole’s function for the MomL secretion capacity in iron deficient condition. (**C**) Indole’s function for the MomL secretion capacity in H_2_O_2_ condition.

**Figure 4:**
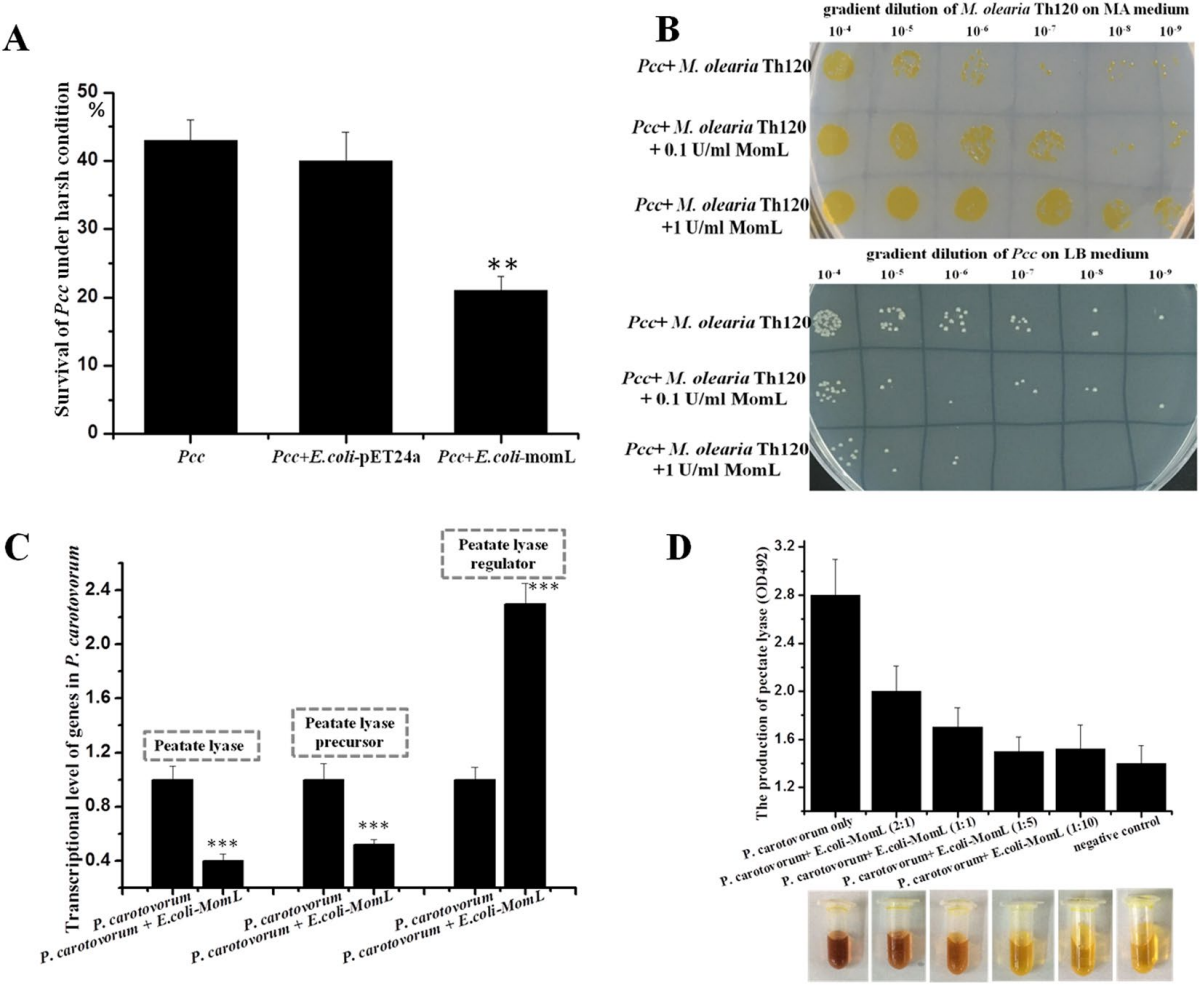
Co-cultivation assays of different bacterial species. (**A**) *P. carotovorum* subsp. *carotovorum* (*Pcc*) and *E. coli-momL* co-cultivation. *E. coli* and *E. coli*- pET24a are used for the negative control. (**B**) *Pcc* and *Muricauda olearia* Th120 co-culture on 10% TSB medium containing 2 mM H_2_O_2_. After culturing, the cultures were serially diluted and spread onto MA and LB plates, separately (MA and LB were medium for *M. olearia* Th120 and *Pcc* respectively. What is more, marine bacterium *M. olearia* Th120 can not survive on LB medium, while, *Pcc* can not live on MA medium). (**C**) Transcriptional analysis of pectate lyase genes of *M. olearia* Th120. (**D**) Detection of pectate lyase production.

MomL secretion ability in different iron conditions was also studied using the *C. violaceum* mutant CV026 as an indicator strain. MomL secretion decreased in an environment of adequate iron nutrition, whereas it was enhanced in iron deficient conditions (Fig. 3A). In iron deficient condition, MomL secretion increased with the addition of indole, which increased 1.2-fold in the presence of 10 and 50 μM indole. At a concentration of 200 μM indole, MomL secretion increased approximately 2-fold in iron deficient condition (Fig. 3B). Besides, under H_2_O_2_ condition, indole could also increase MomL secretion significantly (Fig. 3C). Because of MomL known as AHL lactonase, to analyze whether the AHL lactonase activity plays role in H_2_O_2_ condition, co-cultivation assays of different bacterial species were performed. *P. carotovorum* subsp. *carotovorum* (*Pcc*), once classified as *Erwinia carotovorum* and regarded as a ubiquitous plant pathogen producing virulence factors through the induction of the signal molecule AHL, was used in this experiment^34^. In 10% TSB medium with 2 mM H_2_O_2_, the survival of *Pcc* were 43% and 40% when *Pcc* cells were cultured alone or co-cultured with *E. coli*-pET24a, respectively. Because the survival of *Pcc* was calculated by formula Numbers_LB_ − Numbers_LB+Km_ (method part for detailed information), in order to exclude the possibility of spontaneous Km antibiotic resistance of *Pcc*, control experiment was performed. The result indicated that Km is completely lethal for *Pcc* (Table S2). However, the *Pcc* survival was significantly reduced to 21% when the cells were co-cultured with *E. coli*-*momL*, the *E. coli* strain containing heterologous gene *momL* (Fig. 4A). Moreover, *Pcc* and *M. olearia* Th120 co-culture assays were also performed. *Pcc* had a similar survival with *M. olearia* Th120 under H_2_O_2_ conditions. However, adding exogenous purified MomL protein led to a decreased survival of *Pcc*, and correspondingly, an increased survival of *M. olearia* Th120. As a control assay, boiled inactived MomL had no function (Fig. 4B and Table S3). Transcriptional analysis indicated that the exogenous MomL protein can significantly reduce the expression and the production of the pectate lyase gene, the major virulence factor of *Pcc* (Fig. 4C and D). To study the effect of MomL on pectate lyase expression, four related genes (pectate lyase gene *pel*, pectate lyase precursor 1 gene *pelp1*, pectate lyase precursor 2 gene *pelp2* and pectin lyase regulator gene *pelr*) were detected by real-time PCR. The expression levels of *pel*, *pelp1* and *pelp2* decreased significantly in the presence of MomL. However, the regulator gene *pelr* was up-regulated, leading to speculation that *pelr* plays a negative regulatory role in pectin lyase expression (Fig. 4C).

### Transcriptome analysis of *M. olearia* Th120 under indole conditions

To further analyze the function of indole, we performed transcriptome sequencing of *M. olearia* Th120 with and without 200 μM exogenous indole. Illumina deep sequencing produced approximately 38 M total clean reads from the 5.5 Gbp clean sequence data for each sample. Around 99% of the clean reads had quality scores over the Q20 value. Over 97.9% of the clean reads were mapped to the reference genome (PRJNA244114). In total, 2,885 genes were detected and quantified among all of the samples.

**Figure 5:**
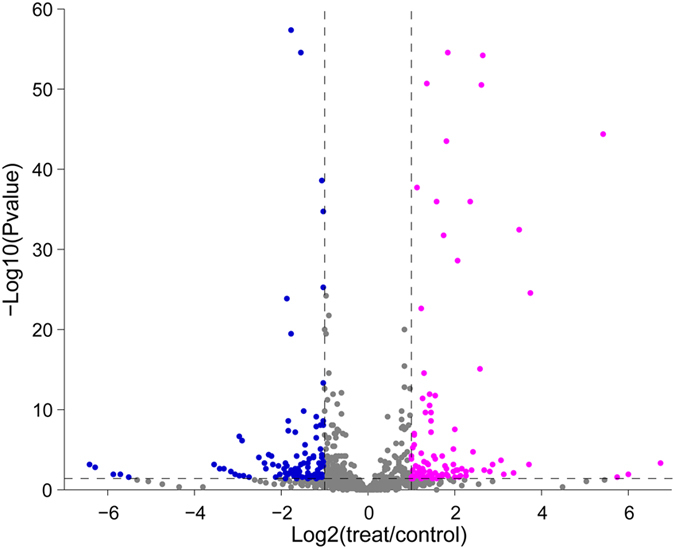
Volcano plots of the overall scatter of gene transcription of *M. olearia* Th120 (with or without indole treatment). P value < 0.05 and |log2 (foldchange)| > 1 were selected as the criteria to filter genes that were significantly regulated (down-regulated genes blue dots, up-regulated genes pink dots).

To test the reliability of the transcriptome assay, we randomly chose 20 genes to perform real-time PCR and found that compared with the transcriptome data, the *R* ^*2*^ value (Pearson correlation coefficient) was 0.9185 (Fig. S4), indicating a good correlation. Moreover, consistent with real-time PCR assay, *suf-momL* gene cluster and *oxyR1* were identified as indole-induced genes in the transcriptome assay (Table S4). Volcano plots, which integrated both the *P* value and fold change of each transcript, were constructed to present the general scattering of the transcripts and filter the differentially expressed genes for indole treatment. Genes regulated by indole were pooled together (Fig. 5, pink and blue color). A total of 206 genes were regulated more than 2-fold by exogenous indole (P > 0.05), among which 104 genes were up-regulated and 102 were down-regulated.

**Figure 6:**
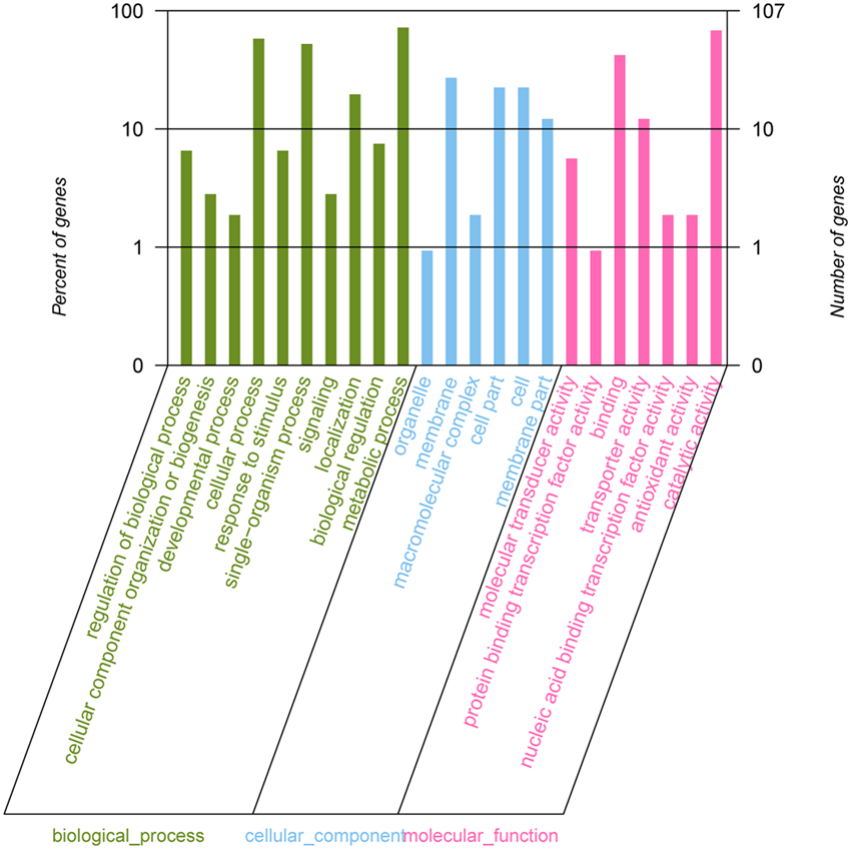
Functional categories of the regulated genes, broadly separated into ‘biological process’, ‘cellular component’, and ‘molecular function’, based on Gene Ontology.

The differentially expressed genes were functionally annotated after being input into the GO analysis and were divided into three major categories: biological process, molecular function and cellular component (Fig. 6). For the sample treated by indole, the most affected biological process were associated with cellular process and metabilic process. Interestingly, multiple genes for response to stimulus and signaling were also affected by indole in *M. olearia* Th120. Furthermore, for cellular component, the significantly regulated part were membrane and membrane-related part. For molecular function, transporter and binding activity were mainly affected by indole. The KOBAS analysis revealed the enriched KEGG pathways under indole treatement (Table S5). Four gene categories, that is, lysine biosynthesis, vancomycin resistance, C5-branched dibasic acid metabolism and two-component system were significantly enriched.

Besides KEGG and GO analysis, our transcriptome results also revealed that five TonB system genes responded to indole, and all of them were up-regulated. Among them, four were noted as TonB-dependent receptors, and their changes in expression ranged from 2 to 8 times the expression levels of indole-free samples. One TonB protein-coding gene was up-regulated 6.5-fold (Table S6). TonB systems can function in iron acquisition. Additionally, 24 membrane proteins, containing transport systems and two component regulatory system proteins, were regulated by indole, among which 21 were up-regulated and only 3 were down-regulated.

## Discussion

*M. olearia* Th120, which was previously isolated from the body surface mucus of *Paralichthys olivaceus*, produces enzymes capable of degrading signal molecules. Located in the genome of *M. olearia* Th120, *momL* encodes an AHL lactonase. The location of the *momL* gene is unique, as it is located within the iron-sulfur protein-coding *suf* genes, with *momL* inserted between *sufB* and *sufC*. The combination of QQ gene(s) and stress-response gene(s) has not previously been observed. The location of the *momL* gene does not disrupt any of the genes in the operon, suggesting that the *momL* location is an adaptation mechanism and not a random insertion. In addition, the *momL* gene is co-transcribed with *suf* under iron deficient and H_2_O_2_ conditions. It is promising to further study whether the AHL lactonase role or other novel function of MomL is needed for the phenotypes. Based on this study, one possible reason is that MomL is secreted into extracellular spaces to degrade the AHL signal molecules of competitors and we speculate that this mechanism might possibly damage the competitors’ QS systems, ultimately decrease the virulence factor “weapon” production and the survival of the competitor. We are constructing MomL mutant with no enzymatic activity using directed evolution strategy to explore the involved mechanism. Moreover, the results above also suggest that *suf* and *momL* are not combined together randomly, but rather, that this special arrangement allows two otherwise unrelated survival mechanisms to be activated by the same environmental stimuli, enhancing cell survival. However, because of the lack of genetic operation system of *M. olearia* Th120, we can not deleted *momL*, and there is currently no direct evidence to prove how *momL* contributes to increased survival of cells under H_2_O_2_ or iron limitation stress. Besides, lacking of *Pcc* genetic operation system at this stage make us unable to delete related genes and unclear about how quorum quenching enzyme MomL decreased the survival of *Pcc*. We are undergoing these experiments in our group now.

**Figure 7:**
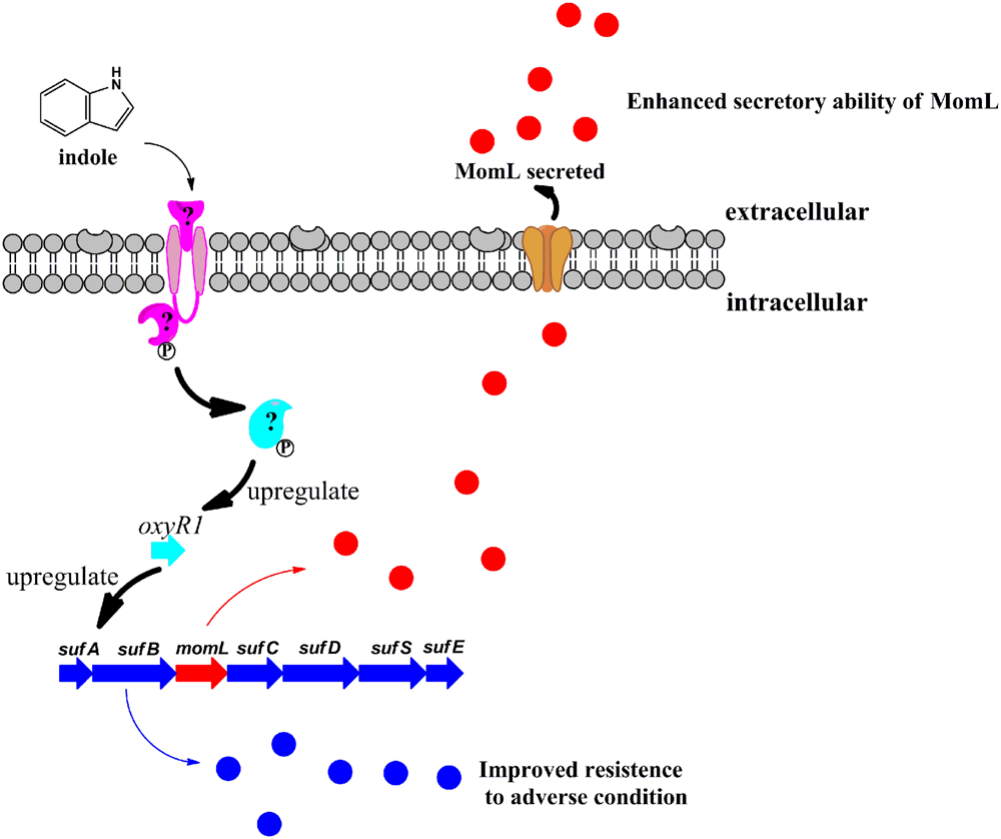
Schematic diagram of the indole and *suf*-*momL* signaling cascade pathway.

The QQ activity of *M. olearia* Th120 can be used in biocontrol for the control and prevention of aquatic animal diseases^17^. As MomL expression is low in cells, it is important to improve its expression for future applications. Signal molecules regulate a series of genes, affecting the physiological activities of a variety of cell types^31, 35, 36^. Therefore, a variety of signal molecules have been tested for improving MomL expression levels in this study. Interestingly, an well-studied signal molecule, indole, had a notable effect on *suf-momL* transcription. Although it is well-studied that indole, as a QS signal molecular, plays important roles in bacteria including biofilm formation, antibiotic resistance and virulence, the mutual regulation between QS and QQ in bacterial is largely unknown. This study demonstrated that indole could not only trigger stress-repsonse genes *suf*, but also regulate QQ enzyme factor *momL*. This is also the first time description that QQ enzyme is triggered by stress factor. Experiments suggested that *oxyR1*, which is regulated by indole, correlated with *suf-momL* expression and MomL secretion. Correspondingly, the addition of indole increases the survival of cells during oxidative stress. We speculate that indole may act as a signaling molecule to regulate the *oxyR1* gene in an unknown way when it senses changes in environmental conditions (Fig. 7). In addition, it is hypothesized that the three *oxyR* genes of *M. olearia* have different functions to allow for more versatile responses to the environment. This discovery of a fundamental response system opens new ways to make this strain more useful as a beneficial bacterium for disease control in the future.

Through transcriptome analyses, it was found that indole regulated more than 200 genes in *M. olearia*; these genes displayed a variety of functions. GO analysis indicated that multiple genes for response to stimulus were affected by indole. It is speculated that these genes were either involved in indole-*suf* signal cascade pathway or among other stress response pathway. Besides, for cellular component, the significantly regulated part were membrane-related genes. Interestingly, most of the membrane proteins were up-regulated under the regulation of indole, including transporters, permeases and TonB systems. Based on this result, we hypothesize that cell membrane proteins play important roles when signaling molecules enter cells. In addition, previous studies have shown that indole could affect the resistance of bacteria to antibiotics^31^. Our transcriptome study showed consistent result. The KOBAS analysis suggested that four KEGG pathways were regulated by indole treatement. Among the four enriched pathways, vancomycin resistance and two-component system were significantly enriched. We suggest that indole can either affect the transport capacity of the cell or change cell permeability to alter the resistance to antibiotics.

## Electronic supplementary material

supporting information
