# Supplementary material for: A novel stress response mechanism, triggered by indole, involved in quorum quenching enzyme MomL and iron-sulfur cluster in Muricauda olearia Th120
Source: Sci Rep. 2017 Jun 26;7:4252. doi: 10.1038/s41598-017-04606-8 (PMC5484670; doi:10.1038/s41598-017-04606-8)
Supplement: Supplementary file 1 — supporting information [file 41598_2017_4606_MOESM1_ESM.pdf]

A novel stress response mechanism, triggered by indole, involved in quorum quenching enzyme MomL and iron-sulfur cluster in *Muricauda olearia* Th120

Yan Wang[1], Hui Li[1], Xinxin Cui[1], Xiao-Hua Zhang[1,2]*

1. College of Marine Life Sciences, Ocean University of China, Qingdao, 266003, China

2. Laboratory for Marine Ecology and Environmental Science, Qingdao National Laboratory for Marine Science and Technology, Qingdao 266071, China

***** *Author to whom correspondence should be addressed; E-Mail: xhzhang@ouc.edu.cn (X.-H.Z.); Tel/Fax: +86-532-82032767.*

Running title: Study of a novel stress response mechanism

Figure S1. The OD_{600} value comparison between with (black) or without indole (transparent) conditions. Error bars represent the standard deviations of three replicates. For statistical analysis, ***/ ** /* means $P<0.001$, $P<0.01$, and $P<0.05$, respectively.

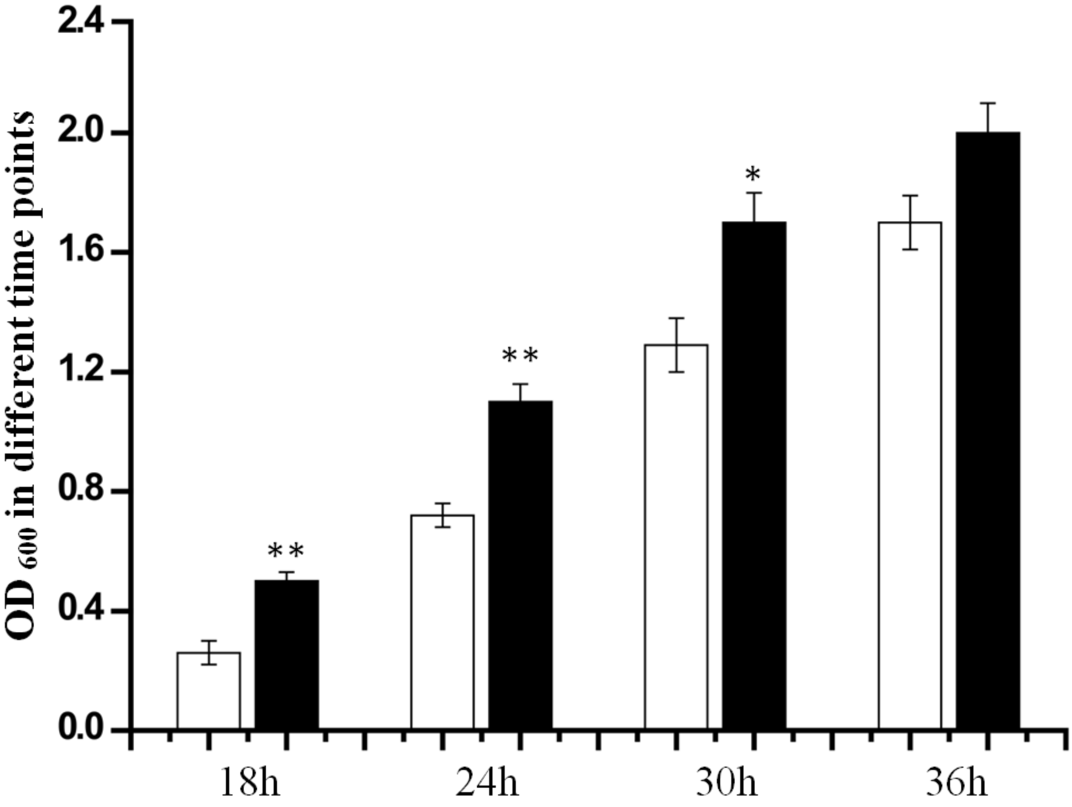

Figure S2. The gene cluster of *suf-momL* of *Muricauda olearia* Th120.

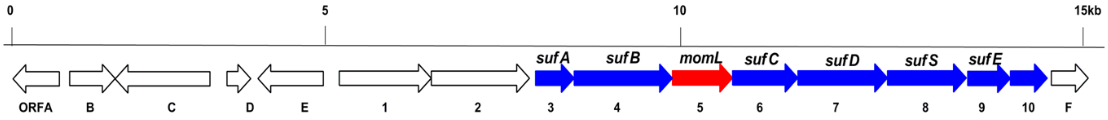

ORF	Blast homolog	#aa
ORFA	member protein	229
ORFB	membrane-associated phospholipid phosphatase	230
ORFC	branched-chain amino acid transport system carrier protein	429
ORFD	hypothetical protein	84
ORFE	thiamine-monophosphate kinase	347
ORF1	regulator/secreted protein	414
ORF2	hypothetical protein	445
ORF3	Fe-S binding protein SufA	109
ORF4	Fe-S metabolism associated SufB	481
ORF5	AHL-lactonase MomL	294
ORF6	ABC transporter ATP-binding protein SufC	249
ORF7	FeS assembly protein SufD	439
ORF8	cysteine desulfurase SufS	408
ORF9	Fe-S metabolism associated SufE	140
ORF10	Fe-S assembly suf system protein	106
ORFF	hypothetical protein	108

Figure S3. Sequence alignment of *oxyR* genes.

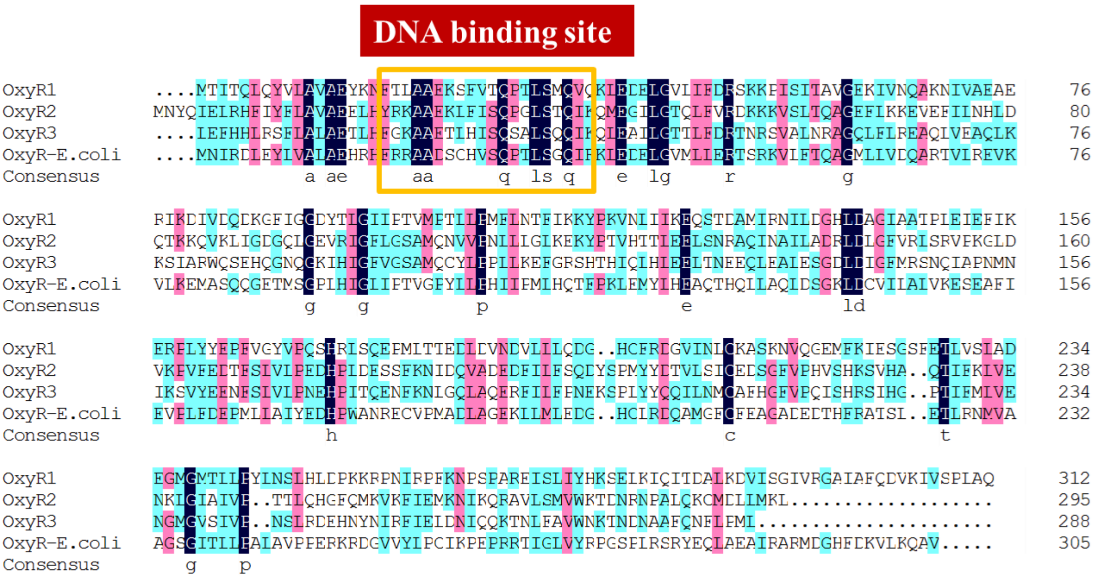

Figure S4. the R^2value (Pearson correlation coefficient) of the transcriptome assay and real-time PCR.

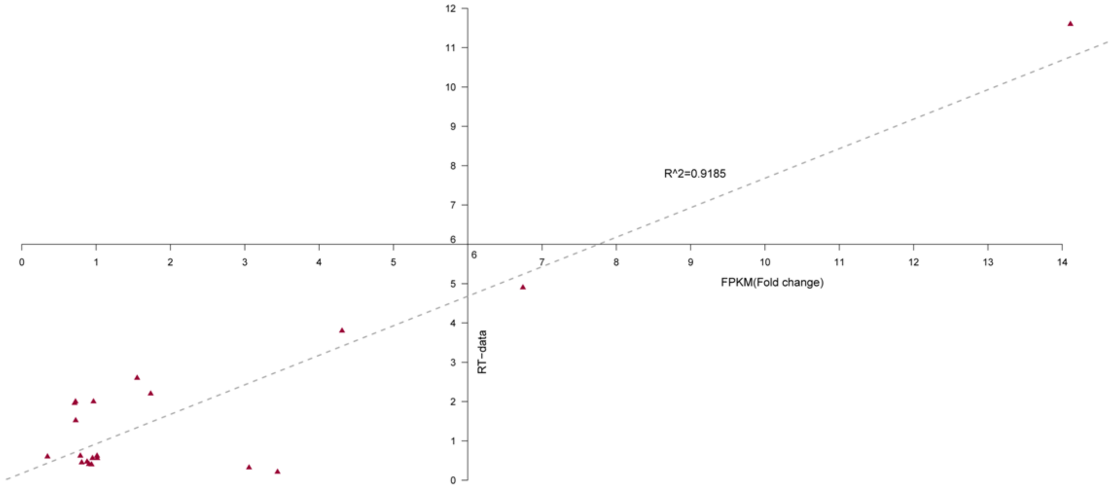

Table S1. Primer information for transcriptional analysis of target genes.

16S-forward	TACGGGAAGAAACCACC
16S-reverse	GCCAGTATCAAAGGCAAT
orfE-forward	GCCTTGCTTCCCTTTC
orfE-reverse	CCACCCAAGTCACTGTTTC
orf1-forward	TGCCTTGGTGGGTCTG
orf1-reverse	TGCCGTGATTACTTGCTT
sufA-forward	TCGGAAACAGCCAAGC
sufA-reverse	CACGGCATTATCCTCAAA
sufB-forward	TGCCGTGGATATTGTCA
sufB-reverse	CCCTTTCGGAATGTAGC
momL-forward	TCTTCCAGAGCCCTTTAC
momL-reverse	GGTTGTCCTCGCTTGTC
sufC-forward	GTCCAGAAAGGCAAGGG
sufC-reverse	CAGAATCGGTCTCATCCA
sufE-forward	TGTCAGAGTAAGGTTTGGGTA
sufE-reverse	AGTTGCTTTACCATACTCACC
orf10-forward	GAAGAAACCACCATAGATACAC
orf10-reverse	GGCAAACTTTCCGCTAC
orfF-forward	CTTGGAAACTGCGCTCTA
orfF-reverse	GATTGGCACCGAGGAA
oxyR1-forward	TGCTACTACAGGACGGACAT
oxyR1-reverse	TTGAGGGCATCGGTTAT
oxyR2-forward	TATCCTACCGTGCATACC
oxyR2-reverse	AAATCTTCATCGGCTACT
oxyR3-forward	TTGGCAATCCGAACACC
oxyR3-reverse	GGTTGGACCGCATAAAA
fur-forward	CGTGTTCACTGCCTTCC
fur-reverse	GCTTGCGTACCAGTTTG
himA-forward	ATCTTTCTTGGGAAATGTAG
himA-reverse	TCAATTCTGCTCCTGCT
himD-forward	CTTGGAGAATGGAGACA
himD-reverse	TTACGCCTTCTACAAAA

Table S2. Survival rate of *Pcc* under Km condition.

Condition	Survival rate of *Pcc*
LB+20 μg/mL Km	0
LB+25 μg/mL Km	0
LB+50 μg/mL Km	0

Table S3. The affect of boiled inactived MomL towards survival rate of *M. olearia* Th120 and *Pcc*.

Condition	Survival rate of *M. olearia* Th120	Survival rate of *Pcc*
Strains Co-culture	41%	39%
Strains Co-culture with 0.1U/ml inactived MomL	40%	40%
Strains Co-culture with 1U/ml inactived MomL	43%	42%

Table S4. List of *suf* related genes regulated by indole in the transcriptome assay.

Gene description	Fold change
sufA-sufE	5.1-8.7
momL	6.3
oxyR1	4.1

Table S5. Significantly enriched pathways and mapped genes regulated by indole based on KEGG pathway analysis.

**gene categories**	***p* value**	**enrich DEGs**	**NR function**	**KEGG function**	**KEGG detailed function**
Lysine biosynthesis (ko00300)	0.0184	*gene301*	diaminopimelate epimerase	K01778|*dapF*	K01778(diaminopimelate epimerase)
		gene1097	UDP-N-acetylmuramoylalanyl-D-glutamyl-2,6-diaminopimelate--D-alanyl-D-alanyl ligase	K01929|*murF*	K01929(UDP-N-acetylmuramoyl-tripeptide--D-alanyl-D-alanine ligase)
		gene2657	UDP-N-acetylmuramoyl-L-alanyl-D-glutamate--2, 6-diaminopimelate ligase	K01928|*murE*	K01928(UDP-N-acetylmuramoyl-tripeptide--D-alanyl-D-alanine ligase)
		gene3005	aspartate kinase	K00928|*lysC*	K00928(aspartate kinase)
Vancomycin resistance (ko01502)	0.0247	*gene882*	D-alanyl-D-alanine dipeptidase	K08641|*vanX*	K08641(D-alanyl-D-alanine dipeptidase)
		gene1097	UDP-N-acetylmuramoylalanyl-D-glutamyl-2,6-diaminopimelate-D-alanyl-D-alanyl ligase	K01929|*murF*	K01929(UDP-N-acetylmuramoyl-tripeptide--D-alanyl-D-alanine ligase)
		gene2656	phospho-N-acetylmuramoyl-pentapeptide-transferase	K01000|*mraY*	K01000(phospho-N-acetylmuramoyl-pentapeptide-transferase)
C5-Branched dibasic acid metabolism (ko00660)	0.0323	*gene160*	3-isopropylmalate dehydrogenase	K00052|*leuB*	K00052(3-isopropylmalate dehydrogenase)
		gene676	succinyl-CoA synthetase subunit beta	K01903|*sucC*	K01903(succinyl-CoA synthetase beta subunit)
		gene170	acetolactate synthase large subunit	K01652|E2.2.1.6L, *ilvB*, *ilvG*, *ilvI*	K01652(acetolactate synthase I/II/III large subunit)
Two-component system (ko02020)	0.0577	*gene1134*	LuxR family transcriptional regulator	K11618|*liaR*	K11618(two-component system, NarL family, response regulator LiaR)
		gene1255	acriflavin resistance protein	K07787|*cusA*, *silA*	K07787(Cu(I)/Ag(I) efflux system membrane protein CusA/SilA)
		gene2036	copper/silver resistance-related transport membrane protein	K07787|*cusA*, *silA*	K07787(Cu(I)/Ag(I) efflux system membrane protein CusA/SilA)
		gene882	D-alanyl-D-alanine dipeptidase	K08641|*vanX*	K08641(D-alanyl-D-alanine dipeptidase)

Table S6. List of TonB transfer system genes regulated by indole.

Gene description	Fold change
TonB-dependent receptor-1	2.3
TonB-dependent receptor-2	3.4
TonB-1	6.5
TonB-2	7.8
TonB-3	7.2
